# Uncommon Presentation of COVID-19 With Long Incubation Period and Only Gastrointestinal Symptoms in a Fully Vaccinated Patient: Is There a Relation?

**DOI:** 10.7759/cureus.16028

**Published:** 2021-06-29

**Authors:** Ahmed S Radhi

**Affiliations:** 1 Internal Medicine, Faculty of Medicine, King Abdulaziz University, Rabigh, SAU

**Keywords:** covid-19, post vaccine, gastrointestinal, long incubation, unusual

## Abstract

Coronavirus disease 2019 (COVID-19) is a respiratory infectious disease that caused a worldwide pandemic in December 2019. It affected millions of people across the world and forced nations to lockdown their borders and implement curfews to control the spread of the disease. Unusual manifestations of the disease should be reported and be kept in mind among physicians. We report a case of a 33-year-old man who was fully vaccinated with two doses of Pfizer vaccine and presented with fever, epigastric pain, and diarrhea without any respiratory symptoms. The patient had a longer than usual incubation period of 16 days without reporting any signs of respiratory infection for the period of his infection. His laboratory investigations and chest X-ray were all normal. The patient did not require any treatment and his COVID-19 infection lasted for 10 days. Clinicians should understand that post-vaccination COVID-19 cases might present with a longer incubation period than usual and might present with gastrointestinal symptoms as the sole complaint.

## Introduction

Coronavirus disease 2019 (COVID-19) emerged in December 2019 in China which caused a new pandemic affecting more than one billion people across 216 countries [1]. The median incubation period is 5.1 days, and 97.5% of the symptoms present within 11.5 days of the infection [2]. These imply that less than 1% of patients will develop symptoms after 14 days of active monitoring or quarantine [2]. One year later, multiple vaccines emerged and the first mass vaccination program started in early December 2020 [3]. The three main clinical manifestations of COVID-19 are fever (57.93%), cough (54.21%), and dyspnea (30.82%) [4]. Gastrointestinal symptoms are less common but are more difficult to recognize as part of a COVID-19 syndrome. Gastrointestinal involvement such as abdominal pain, nausea, vomiting, and diarrhea have been recently reported in the literature with diarrhea (9.59%) being the most common gastrointestinal symptom [4]. Diarrhea occurs secondary to the interaction between angiotensin-converting enzyme 2 (ACE2) and 2019-nCoV cell entry receptor ACE2 [5]. It was found that the ACE2 expression is 100-fold higher in the gastrointestinal system than in the respiratory system [5]. Recent studies showed that 2019-nCoV RNA can be detected in stool samples, confirming fecal-oral transmission of COVID-19 reaching up to 25 days after respiratory symptoms subside, even after nasal swabs were negative [6]. There are large clinical studies that were done in China on gastrointestinal symptoms and detection of the virus in feces. Jin et al. investigated 74 patients infected with severe acute respiratory syndrome coronavirus 2 (SARS-CoV-2) with gastrointestinal symptoms such as diarrhea, nausea, and vomiting [7]. Up to 28% of those with gastrointestinal symptoms did not have respiratory symptoms. We report a case of COVID-19 that presented initially with gastrointestinal symptoms namely abdominal pain and diarrhea as the primary and only symptom at the time of presentation.

## Case presentation

**Figure 1:**
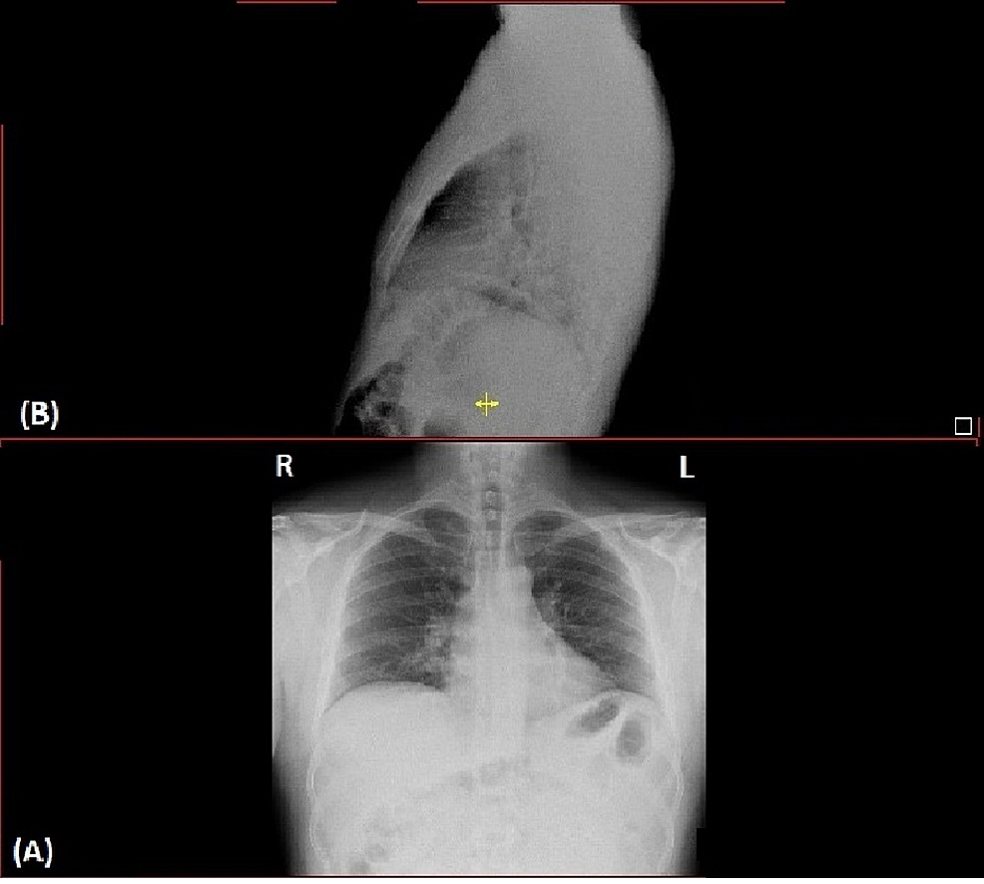
Chest X-ray: (A) lateral view and (B) anteroposterior view. R: Right, L: Left

A 33-year-old male patient had his first dose of Pfizer vaccine on the 8th of January 2021 and the second dose on the 16th of February 2021. He reported an incident of close contact with a COVID-19 patient on the 10th of April 2021 with no other contact since. The patient was following social distancing and mask-wearing in public. He reported that he had two nasal swabs on the second and fifth day after exposure which was negative. Regarding his medical history, he has gastroesophageal reflux disease (GERD) and is on pantoprazole. He does not smoke, drinks alcohol, or use illicit drugs and he had no known history of allergies. On the 26th of April 2021, he presented to the emergency department (ED) with epigastric pain, nausea, diarrhea, and a low-grade fever of 38.2°C. Furthermore, there were no respiratory symptoms or neurological symptoms reported or seen such as cough, shortness of breath, fatigue, muscle pain, or tiredness. His oxygen saturation (SaO2) was 98%, temperature 38.2˚C, respiratory rate 18 breaths/minute, and heart rate 105 beats/minute. Meanwhile, his laboratory investigation including a complete blood count which showed a white blood cell (WBC) count of 11.6x109/L, hemoglobin 13.9 g/dL, platelet 193 109/L, neutrophil 84.6 cells/µL, lymphocytes 1.2 cells/µL, and chest X-ray was normal (Figure 1).

He had a specimen for real-time reverse-transcriptase-polymerase-chain-reaction (rRT-PCR) assay on the same day which was positive for COVID-19. He received IV paracetamol and was sent home where he was quarantined. His fever lasted for 24 h but his abdominal pain and diarrhea lasted for seven days and was resolved. He completed a 10-day quarantine with no further complications or any signs of respiratory symptoms.

## Discussion

COVID-19 presenting gastrointestinal symptoms has been increasing dramatically in patients with symptoms such as loss of appetite, nausea, vomiting, diarrhea, and abdominal pain [8]. Our patient presented only with fever, epigastric pain, and diarrhea which can mimic many gastrointestinal diseases such as gastroenteritis, esophagitis, and peptic ulcer disease [8]. History of GERD with a positive status of vaccine should not eliminate the possibility of contracting COVID-19. Infection cases post-vaccination have been reported to occur in a less aggressive form of the disease [9]. Menni et al. studied the risk of infection after COVID-19 vaccination and reported that those older than 55 years of age had a much lower risk of getting infected after the vaccine. In addition, patients with multiple comorbidities had a higher chance of getting infected than those with no comorbidities post-vaccination [9]. However, our patient did not have any comorbid disease and was 33 years old. Moreover, he presented with a longer incubation period of 16 days than the usual 2-14 days. The unusually long incubation period and the unusual presenting symptom with only gastrointestinal symptoms alone can be hypothesized to two facts. The first can be due to the vaccination history of the patient making the immune system recognize the virus making it less infectious. The second one is that it can be just an unusual presentation of COVID-19. To make any connection between the vaccine and the longer incubation period, more studies are required to understand the link. Clinicians should stay alert on the presentation of such cases as vaccination status does not necessarily mean the patient is not susceptible to infection. It might cause delayed and uncommon presentations. Therefore, quarantining such cases is necessary to reduce social infection until a COVID-19 result comes back negative.

## Conclusions

COVID-19 can still infect those vaccinated to a lesser extent. However, unusual symptoms such as presenting with only gastrointestinal symptoms and no respiratory symptoms are unusual. In addition, the incubation period of the virus can be longer in such presentations. Clinicians should be on alert concerning such cases and ask the patient to quarantine immediately until the COVID-19 test comes back negative.
